# Combinatorial effects of a novel SHV-248 variant, NDM-5, and *ompK35* deficiency drive high-level cefiderocol resistance in *Klebsiella pneumoniae*

**DOI:** 10.1128/spectrum.00026-26

**Published:** 2026-04-30

**Authors:** Chuwen Zhao, Yuxuan Liu, Hanxu Hong, Yun Cao, Xinyue Dai, Qianbin Dai, DanDan Wei, Yang Liu

**Affiliations:** 1 Department of Clinical Laboratory, The First Affiliated Hospital, Jiangxi Medical College, Nanchang University 117970, Nanchang, China; 2 First Clinical Medical College, Nanchang University 47861, Nanchang, China; 3 School of Public Health, Jiangxi Medical College, Nanchang University, Nanchang, China; 4 Jiangxi Medical Center for Critical Public Health Events, The First Affiliated Hospital, Jiangxi Medical College, Nanchang University 117970, Nanchang, China; 5 China-Japan Friendship Jiangxi Hospital, National Regional Center for Respiratory Medicine, Nanchang, China; Newcastle University, Newcastle Upon Tyne, United Kingdom

**Keywords:** cefiderocol, porin deficiency, resistance mechanism, carbapenem-resistant, *Klebsiella pneumoniae*, *bla*_SHV-248_, metallo-β-lactamase

## Abstract

Cefiderocol holds significant therapeutic value against metallo-β-lactamase-producing Enterobacterales. However, reduced susceptibility to cefiderocol in NDM-producing strains is increasingly observed. This study elucidated the mechanism of high-level cefiderocol resistance in a clinical *Klebsiella pneumoniae* strain (KP5663, ST147-KL64) producing NDM-5, which emerged without prior drug exposure. Whole-genome sequencing identified, alongside *bla*_NDM-5_ and *ompK35*/*ompK36* porin deficiencies, a novel β-lactamase gene variant, *bla*_SHV-248_. Functional characterization revealed that SHV-248 exhibited a moderately higher affinity for cefiderocol (binding energy: −5.15 kcal/mol) and a 1.6-fold greater hydrolytic activity against nitrocefin compared to SHV-12. Consistent with this, heterologous expression of *bla*_SHV-248_, but not *bla*_SHV-12_, increased the cefiderocol minimum inhibitory concentration (MIC) by fourfold, although the MIC remained within the susceptible range. Critically, genetic complementation demonstrated that restoring functional *ompK35* alone reduced the cefiderocol MIC of KP5663 by 16-fold, whereas complementation with *ompK36* had no effect. These findings define a novel synergistic triad wherein the combined, rather than individual, action of *bla*_NDM-5_, the novel variant *bla*_SHV-248_, and porin deficiency (primarily *ompK35*) is necessary and sufficient for high-level cefiderocol resistance.

**IMPORTANCE:**

This study identifies a novel SHV variant and, more importantly, delineates a distinct “key determinant combination” model for cefiderocol resistance. This model does not rely on exceptional enzyme overproduction but on the convergence of three clinically relevant factors: a transmissible *bla*_NDM-5_, a chromosomally evolving SHV variant with incremental efficiency gains, and frequent porin loss. It underscores that, for NDM-producing *Klebsiella pneumoniae*, risk assessment must evolve to evaluate porin status alongside β-lactamase profiles, as even modestly more efficient enzymes like SHV-248 can become decisive factors within a permissive genetic background.

## INTRODUCTION

The emergence and global spread of carbapenem-resistant Enterobacterales (CRE) pose a major threat to public health. The production of carbapenemases is a key mechanism of resistance, which includes class A and class D serine β-lactamases and class B metallo-β-lactamases (MBLs) (1). To address this challenge, several novel β-lactam/β-lactamase inhibitor combinations have been developed. Those with potent activity against serine carbapenemases include ceftazidime-avibactam, meropenem-vaborbactam, and imipenem-relebactam. Although diazabicyclooctane β-lactam enhancers (e.g., zidebactam and nacubactam) and the boronic acid-based broad-spectrum β-lactamase inhibitor taniborbactam show promising potential against MBLs, they have not yet been approved for clinical use (2–4). In China, ceftazidime-avibactam combined with aztreonam remains one of the limited therapeutic options against MBL-producing Enterobacterales (5).

Cefiderocol, a novel siderophore cephalosporin soon to be launched in China, primarily targets penicillin-binding protein 3 (PBP3). It enters the periplasmic space of gram-negative bacteria primarily through the active iron-siderophore uptake systems and, to some extent, via traditional porin channels (6, 7). Its structure confers stability against hydrolysis by many β-lactamases, including MBLs, making it a vital therapeutic option for infections caused by MBL-producing pathogens (7, 8). Unfortunately, epidemiological studies indicate that NDM-producing Enterobacterales generally exhibit lower susceptibility to cefiderocol compared to strains producing other types of MBLs (5, 9). Furthermore, Faxén et al. reported that according to the European Committee on Antimicrobial Susceptibility Testing (EUCAST) criteria, only 41% (25/61) of NDM-producing Enterobacterales isolates were susceptible to cefiderocol (4).

The clinical concern regarding cefiderocol treatment failures driven by NDM-mediated resistance is growing (10). However, the precise molecular mechanisms underlying NDM-mediated cefiderocol resistance are not fully elucidated (10). Proposed explanations primarily involve mutations or deletions in genes related to the siderophore iron-transport system (e.g., *CirA*, *TonB*), mutations in the target PBP3, and the co-production of multiple carbapenemases (11–14). The combined effect of these factors with NDM is considered a key mechanism reducing bacterial susceptibility to cefiderocol. Additionally, Simner et al. demonstrated that during cefiderocol treatment of NDM-producing *Escherichia coli*, bacteria can enhance resistance by increasing *bla*_NDM_ gene copy number and expression levels (15).

Notably, beyond the siderophore system, the roles of traditional porins and other highly efficient β-lactamases are gaining attention. Recent studies suggest that deficiencies in the *ompK35*/*36* porins and tandem amplification of *bla*_SHV-12_ can also reduce bacterial susceptibility to cefiderocol (16, 17). In this study, we identified and characterized a novel SHV variant and uncovered a new mechanism of NDM-driven cefiderocol resistance: the combined impact of *ompK35* deficiency, *bla*_NDM-5_, and the novel SHV variant *bla*_SHV-248_ confers high-level cefiderocol resistance in *Klebsiella pneumoniae*. This finding indicates that porin deficiency and the presence of high-hydrolytic-efficiency β-lactamases are critical factors contributing to cefiderocol resistance in NDM-producing *K. pneumoniae*, offering a new perspective for understanding the theoretical framework of this resistance mechanism.

## MATERIALS AND METHODS

### Bacterial isolate and antimicrobial susceptibility testing

The *Klebsiella pneumoniae* isolate KP5663 was obtained from a clinical patient sample at a tertiary hospital in China in 2025. Species identification was confirmed by matrix-assisted laser desorption/ionization time-of-flight mass spectrometry (Bruker Daltonics). Antimicrobial susceptibility testing was performed using the VITEK-2 system (bioMérieux, Lyon, France), and results were interpreted according to the Clinical and Laboratory Standards Institute guidelines (18). The minimum inhibitory concentration (MIC) of cefiderocol was determined by the broth microdilution method in iron-depleted cation-adjusted Mueller-Hinton broth, with susceptibility interpreted per the EUCAST criteria (https://www.eucast.org/). *Escherichia coli* ATCC 25922 was used as the quality control strain. For inhibitor experiments, the final concentrations of avibactam sodium and ethylenediaminetetraacetic acid (EDTA) were fixed at 4 mg/L and 0.4 mM, respectively. For the inhibitor synergy test, a bacterial suspension of KP5663 (0.5 McFarland standard) was evenly spread onto Mueller-Hinton agar plates. Four cefiderocol discs (30 μg/disc) were placed on the inoculated agar. Subsequently, three discs were supplemented, respectively, with EDTA (292 μg/disc), avibactam (AVI) (20 μg/disc), or a combination of both inhibitors (EDTA 292 μg/disc and avibactam 20 μg/disc). After incubation at 37°C for 18–20 h, the inhibition zone diameters were measured.

### Bioinformatic analysis

Genomic DNA was extracted from a single colony using the TIANamp Bacterial DNA Kit (TianGen Biotech, Beijing, China). Whole-genome sequencing was performed using the NovaSeq 6000 platform (Illumina, San Diego, CA, USA) for short-read sequencing and the QPursue-6khex platform (Qitan Tech) for long-read sequencing. The sequencing data were assembled *de novo* using Unicycler v0.4.5. Genome annotation was conducted using Bakta v1.9.2. Sequence type (ST) and capsular serotype were analyzed via the Pathogenwatch online platform (https://pathogen.watch/). Plasmid replicon types were identified using PlasmidFinder v2.1. A phylogenetic tree was constructed based on the neighbor-joining method with MEGA 12 software and visualized using iTOL (https://itol.embl.de/). Genomic linear comparisons and circular map alignments were generated using Easyfig 2.2.5 and BLAST Ring Image Generator, respectively.

### Cloning and complementation

The genes for *bla*_SHV-248_, *bla*_NDM-5_ (with their native promoters), *bla*_SHV-12_ (with its promoter), and the full-length *ompK35* and *ompK36* were amplified from templates KP5663, the *Klebsiella pneumoniae* clinical strain OP135 (accession number: SAMN48387653), and the *K. pneumoniae* reference strain ATCC 700603, respectively. These fragments were individually cloned into the pBAD33 vector using the ClonExpress II One Step Cloning Kit (#C112-01, Vazyme, Nanjing Vazyme Biotech Co., Ltd.) and transformed into *E. coli* DH5α competent cells, with positive clones selected on chloramphenicol (50 mg/L) plates to obtain DH5α/SHV-248, DH5α/SHV-12, and DH5α/NDM-5 recombinants. Subsequently, the respective plasmids were electroporated into different backgrounds: pBAD33-SHV-248 and pBAD33-NDM-5 were introduced into *K. pneumoniae* ATCC 700603 to generate recombinant strains ATCC 700603/SHV-248 and ATCC 700603/NDM-5, while pBAD33-*ompK35* and pBAD33-*ompK36* were introduced into the original strain KP5663 to generate complementation strains KP5663/*ompK35* and KP5663/*ompK36*, with all transformants selected on hygromycin (100 mg/L) plates. All constructed strains were verified by PCR and sequencing. Cefiderocol susceptibility testing for all strains harboring pBAD33-derived plasmids was performed in iron-depleted broth supplemented with 0.1% arabinose.

### RT-qPCR assay

As described in a previously published method (19), total RNA was extracted from bacterial cultures using the RNA prep Pure Cell/Bacteria Kit (Tiangen, China). Reverse transcription was performed to generate cDNA. Quantitative real-time PCR (qPCR) was carried out to assess the relative expression levels of the *bla*_SHV_ and *bla*_NDM_ genes. The *16S rRNA* gene served as the endogenous control for normalization. The relative expression levels were calculated using the 2^−ΔΔCT^ method. Each sample was analyzed in triplicate.

### Conjugation assay

Plasmid conjugation experiments were performed as described previously (20). KP5663, harboring the *bla*_NDM-5_ plasmid, was used as the donor strain, and sodium azide-resistant *E. coli* J53 served as the recipient strain. After co-culture, putative transconjugants were selected on double-antibiotic plates containing 2 mg/L meropenem and 200 mg/L sodium azide and subsequently verified by PCR.

### β-lactamase activity assay

The enzymatic activities of SHV-248 and SHV-12 were assessed using crude cell extracts, as previously described with modifications (21). Briefly, recombinant *E. coli* strains harboring the respective *bla*_SHV_ genes were cultured to mid-logarithmic phase in LB broth. Bacterial cells were harvested by centrifugation, washed, and resuspended in 0.01 M PBS (pH 7.4). Cell disruption was performed by sonication on ice using an ultrasonic cell disruptor (Yetuo, China) with multiple short pulses for a total of 10 min. The lysate was centrifuged at 12,000 × *g* for 60 min at 4°C to remove cell debris, and the supernatant was collected as the crude enzyme extract. Hydrolysis of nitrocefin (100 μM) was monitored by measuring the increase in absorbance at 486 nm for 15 min using a Varioskan Lux microplate reader (Thermo Fisher Scientific, USA). One unit of enzyme activity was defined as the amount of enzyme required to hydrolyze 1 nmol of nitrocefin per minute.

### Molecular modeling and docking

Protein modeling and molecular docking were performed as described previously (22). The protein structure of SHV-11 (accession number: 6NFD) was obtained from the RCSB Protein Data Bank. The three-dimensional structures of SHV-12 and SHV-248 were predicted using AlphaFold3. Molecular docking was performed using AutoDock 4 (23), and the results were visualized using PyMOL.

### Statistical analysis

All statistical analyses were performed using GraphPad Prism v10.1.2. Comparisons of gene expression levels between groups were analyzed using the Wilcoxon rank-sum test. A *P* value of < 0.05 was considered statistically significant.

## RESULTS

### Identification of a novel SHV variant in a cefiderocol-resistant *K. pneumoniae* ST147-KL64 isolate

In 2025, a cefiderocol-resistant *Klebsiella pneumoniae* strain, KP5663, was isolated from a patient in a hospital in Jiangxi Province. This strain demonstrated resistance to most β-lactam antibiotics (including ceftazidime, cefepime, and carbapenems) and quinolones, but remained susceptible to aztreonam and amikacin. Notably, cefiderocol had not yet been introduced into clinical use locally, indicating that this strain exhibited natural resistance without prior antibiotic induction.

**Fig 1:**
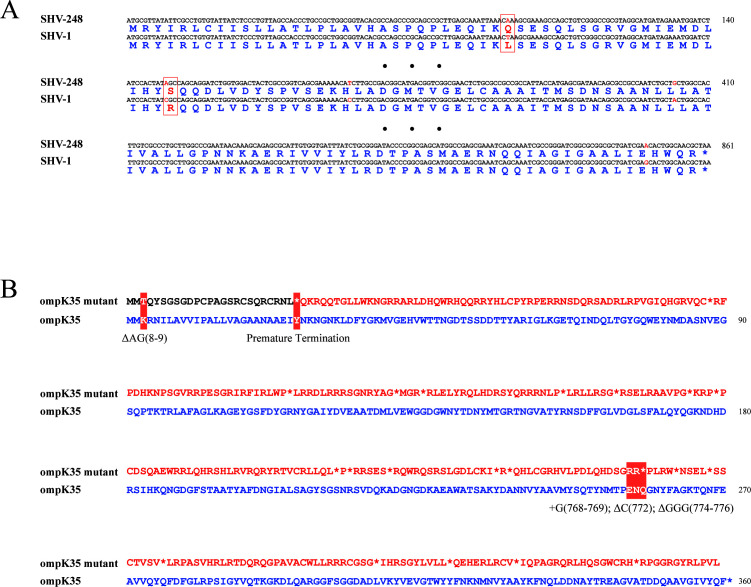
Sequence variations of the novel *bla*_SHV-248_ variant and truncated *ompK35* in strain KP5663. (**A**) Sequence alignment of *bla*_SHV-1_ and *bla*_SHV-248_. Nucleotide and corresponding amino acid substitutions are highlighted in red and boxed. Only partial sequences flanking the mutation sites and the terminal regions are shown; omitted internal sequences are indicated by “• • •”. (**B**) Protein sequence alignment of full-length *ompK35* and the truncated *ompK35* variant. Altered amino acids and the premature transcriptional termination site are indicated by white text on a red background. Δ, nucleotide deletion; +, nucleotide insertion. Numbers in parentheses indicate nucleotide positions.

To elucidate the mechanism underlying high-level cefiderocol resistance in this *K. pneumoniae* isolate, we performed nanopore-based whole-genome sequencing on KP5663. The results showed that the KP5663 chromosome was 5,421,280 bp in length and carried six plasmids ranging in size from 216,969 bp to 2,045 bp. Analysis of the assembled sequence via Pathogenwatch revealed that the strain belonged to sequence type 147 (ST147) with capsular serotype KL64. While ST147 is not the most prevalent clone in China, it has been reported as a high-risk clone spreading globally, often carrying extended-spectrum β-lactamases. Resistance gene analysis identified 16 resistance genes in KP5663, including *bla*_NDM-5_, *bla*_OXA-1_, and a novel SHV variant, *bla*_SHV-248_. Whole-genome sequencing confirmed that *bla*_SHV-248_ is located on the chromosome, while *bla*_NDM-5_ is plasmid-borne. Sequence alignment revealed that *bla*_SHV-248_ differed from *bla*_SHV-1_ by five nucleotide substitutions, resulting in two amino acid changes at positions 31 and 94 (Fig. 1A; Fig. S1). Phylogenetic analysis based on single-nucleotide polymorphisms indicated a close relationship with the clinically common SHV-11, suggesting that it may have evolved from SHV-11 (Fig. S2). We found no evidence of gene amplification or promoter-region mutations for *bla*_SHV-248_ in the sequencing data. Besides β-lactamases, sequencing also revealed abnormalities in the outer membrane porins: the *ompK36* gene was completely absent, while a double nucleotide deletion (positions 9 and 10) in the *ompK35* gene caused a frameshift and generated a premature stop codon at amino acid position 26, resulting in a truncated protein (Fig. 1B). As no definitive mutations or premature stop codons were identified within the coding sequences or predicted promoter regions in key siderophore uptake-related genes (e.g., *cirA*, *fiu*, *fepA*, *iutA*, *sitC*, *apbC*, *fepG*, *fepC*, *fetB*, *yicI*) in KP5663, we hypothesized that the high-level cefiderocol resistance in KP5663 likely resulted from the combined action of *bla*_SHV-248_, *bla*_NDM-5_, and porin deficiency.

### SHV-248 exhibits moderately enhanced hydrolytic activity against cefiderocol than SHV-12

To characterize the function of SHV-248, we cloned and heterologously expressed it in *E. coli* DH5α using the pBAD33 vector. The MIC of cefiderocol for the *bla*_SHV-248_-expressing recombinant strain increased to 0.064 mg/L, which was at least fourfold higher than that of the empty vector control (MIC: 0.016 mg/L), yet still within the susceptible range (Table 1). Additionally, susceptibility testing against a panel of β-lactams revealed that the strain was resistant to ampicillin, exhibited intermediate susceptibility to ampicillin-sulbactam, and remained susceptible to ceftazidime, cefotaxime, and amoxicillin-clavulanate.

**TABLE 1:** Cefiderocol MICs for various bacterial strains used in this study^*a*^

Species	Strain/plasmid	β-lactamase(s) and porin	MIC (mg/L)
*K. pneumoniae*	KP5663 (clinical isolate)	SHV-248, NDM-5, truncated *ompK35*	128
*E. coli*	DH5α/pBAD33		0.016
*E. coli*	DH5α/pBAD33-SHV-248	SHV-248	0.064
*E. coli*	DH5α/pBAD33-SHV-12	SHV-12	0.016
*E. coli*	DH5α/pBAD33-NDM-5	NDM-5	1
*E. coli*	J53		0.016
*E. coli*	J53/pKP5663-NDM-5	NDM-5	1
*K. pneumoniae*	ATCC 700603/pBAD33	SHV-18	0.25
*K. pneumoniae*	ATCC 700603/pBAD33-SHV-248	SHV-18, SHV-248	0.5
*K. pneumoniae*	ATCC 700603/pBAD33-NDM-5	SHV-18, NDM-5	8
*K. pneumoniae*	KP5663/pBAD33	SHV-248, NDM-5, truncated *ompK35*	128
*K. pneumoniae*	KP5663/pBAD33-*ompK35*	SHV-248, NDM-5, truncated *ompK35*, functional *ompK35* by complementation	8
*K. pneumoniae*	KP5663/pBAD33-*ompK36*	SHV-248, NDM-5, truncated *ompK35*, functional *ompK36* by complementation	128

^*a*^ KP5663 is the parental clinical isolate harboring *bla*_SHV-248_, *bla*_NDM-5_, and truncated *ompK35*. The remaining strains were constructed by transformation, electroporation, or conjugation using DH5α, J53, ATCC 700603, or KP5663 as recipients.

**Fig 2:**
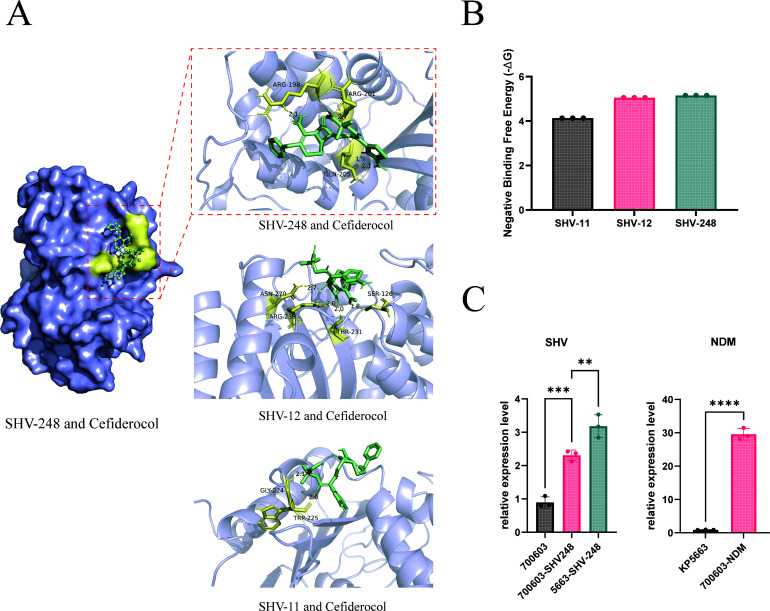
Molecular docking of cefiderocol with SHV enzymes and relative expression of β-lactamases in KP5663 and its transformants. (**A**) Molecular docking visualization of cefiderocol with SHV-11, SHV-12, and SHV-248. Cefiderocol and amino acid residues are shown in green and yellow sticks, respectively. Hydrogen bonds and their lengths are indicated. (**B**) Comparison of the calculated free binding energy between cefiderocol and SHV-11, SHV-12, and SHV-248. (**C**) Relative expression levels of *bla*_SHV_ and *bla*_NDM_ genes in the transformants, along with the reference strains *K. pneumoniae* ATCC 700603 and KP5663. *P < 0.05; **P < 0.01; ***P < 0.001; ****P < 0.0001.

Given that *bla*_SHV-12_ has been reported to mediate high-level cefiderocol resistance via gene tandem amplification, we compared the substrate affinity of SHV-248 with SHV-12 and SHV-11. Molecular docking simulations revealed that the binding energy of SHV-248 with cefiderocol (−5.15 kcal/mol) was more negative than that of SHV-12 (−5.05 kcal/mol) and SHV-11 (−4.13 kcal/mol) (Fig. 2A and B), suggesting that SHV-248 may possess stronger substrate affinity. Concurrently, we determined the hydrolytic activity of SHV-248 and SHV-12 against the broad-spectrum substrate nitrocefin. The results showed that the enzymatic activity of SHV-248 was approximately 1.6-fold higher than that of SHV-12, indicating that SHV-248 intrinsically possesses a higher basal hydrolysis efficiency.

We then expressed *bla*_SHV-12_ in the same system to compare its phenotypic impact. The results demonstrated that, whereas the expression of *bla*_SHV-248_ increased the cefiderocol MIC, the expression of *bla*_SHV-12_ did not alter the MIC compared to the empty vector control (0.016 mg/L) (Table 1). Furthermore, introducing *bla*_SHV-248_ into the *K. pneumoniae* reference strain ATCC 700603 (which constitutively expresses *bla*_SHV-18_) elevated the cefiderocol MIC from 0.25 mg/L to 0.5 mg/L (Table 1). RT-qPCR analysis indicated that the basal expression level of *bla*_SHV-248_ in the original KP5663 strain was higher than its induced expression level in the ATCC 700603 recombinant strain (Fig. 2C). These results collectively indicate that SHV-248 exhibits moderately enhanced hydrolytic activity against cefiderocol compared to SHV-11 and SHV-12. Although its individual expression under the experimental conditions was insufficient to confer clinical resistance, this variant carries the risk of mediating resistance at a clinical level if its expression is elevated (e.g., through gene amplification or promoter mutations).

### NDM-5 and SHV-type enzymes play a collaborative role in cefiderocol resistance

**Fig 3:**
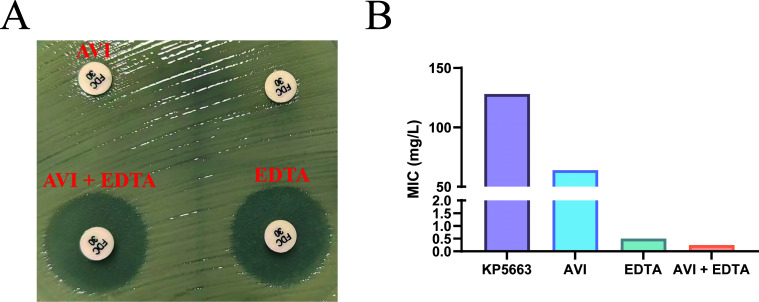
Enzyme inhibitor assays for strain KP5663. (**A**) Results of the disk diffusion assay with enzyme inhibitors. Four cefiderocol discs (30 μg/disc) were placed on the inoculated agar. The upper-left disc was supplemented with avibactam, the upper-right disc contained cefiderocol only (control), the lower-right disc was supplemented with EDTA, and the lower-left disc was supplemented with both avibactam and EDTA. (**B**) Results of the broth microdilution method with enzyme inhibitors.

Although previous studies have indicated that NDM alone cannot confer high-level cefiderocol resistance, its contribution to the resistant phenotype in KP5663 required clarification (24). We evaluated the roles of NDM-5 and SHV-248 using inhibitor synergy tests: the addition of the metallo-β-lactamase inhibitor EDTA drastically reduced the cefiderocol MIC for KP5663 from 128 mg/L to 0.5 mg/L (the inhibition zone diameter increased from 6 mm to 20 mm). Considering the critical influence of iron on cefiderocol susceptibility, when avibactam was added, although no expansion of the inhibition zone was observed on iron-replete agar (Fig. 3A), a decrease in MIC to 64 mg/L was detected in the iron-depleted broth microdilution assay (Fig. 3B). When EDTA and AVI were used in combination, the MIC was further reduced to 0.25 mg/L. This indicates that NDM-5 is one of the primary contributors to high-level cefiderocol resistance, while the presence of SHV-248 further enhances the resistance of KP5663 to cefiderocol.

The *bla*_NDM-5_ gene was located on a 46,132 bp IncX3-type plasmid, pKP5663-NDM. BLAST comparison showed that this plasmid was highly homologous (95% coverage, 99.99% identity) to plasmid pGDQ8D112M-NDM (accession number: NZ_MK628734.1.) from *Klebsiella pneumoniae* GDQ8D112M. One study reported a *cirA*-deficient, NDM-5-producing *K. pneumoniae* strain that was resistant to cefiderocol (MIC > 256 mg/L). Compared to the plasmid pAR8416 (NZ_CP081819.1) from that study, pKP5663-NDM contained an additional 877 bp sequence upstream of the *bla*_NDM-5_ gene (Fig. S3), suggesting potential differences in the expression regulation of *bla*_NDM-5_ on pKP5663-NDM. To directly validate the role of NDM-5, we cloned *bla*_NDM-5_ along with its upstream fragment into the pBAD33 vector and expressed it in different hosts. In *E. coli* DH5α, which does not harbor other significant β-lactamases, the introduction of *bla*_NDM-5_ only increased the cefiderocol MIC from 0.016 mg/L to 1 mg/L. However, introducing *bla*_NDM-5_ into *K. pneumoniae* ATCC 700603, which endogenously expresses *bla*_SHV-18_, elevated the cefiderocol MIC from 0.25 mg/L to 8 mg/L, reaching the resistant level. This suggests that the coexistence of NDM-5 with other β-lactamases (such as SHV) can synergistically lead to cefiderocol resistance. Notably, RT-qPCR analysis showed that the expression level of *bla*_NDM-5_ in the ATCC 700603 recombinant strain was even higher than that in the original KP5663 strain (Fig. 2C), yet the MIC it mediated (8 mg/L) was far lower than that of KP5663 (128 mg/L). Similarly, conjugal transfer of the native plasmid pKP5663-NDM into *E. coli* J53 only resulted in an increase of the cefiderocol MIC to 1 mg/L. This value was identical to that of the recombinant strain DH5α/pBAD33-NDM-5 (1 mg/L), suggesting that plasmid-specific elements beyond *bla*_NDM-5_ did not contribute to the high-level resistance observed in KP5663. This indicates that the expression level of *bla*_NDM-5_ is not the sole determinant of the degree of cefiderocol resistance. Although the coexistence of *bla*_NDM-5_ and *bla*_SHV-248_ partially explains the resistance, the high-level resistance observed in KP5663 suggests the involvement of additional chromosome-derived mechanisms that collectively contribute to mediating high-level cefiderocol resistance.

### High-level cefiderocol resistance in KP5663 results from the combined action of porin deficiency, *bla*_NDM-5_, and *bla*_SHV-248_

Although porin truncation and deficiency are relatively common in *Klebsiella pneumoniae*, particularly in extended-spectrum β-lactamase (ESBL)-producing strains, cases of porin deficiency synergizing with β-lactamases to mediate cefiderocol resistance have been reported. To clarify the specific role of porin deficiency in the resistance of KP5663, genetic complementation of the functional porin genes *ompK35* and *ompK36*, amplified from the reference strain *K. pneumoniae* ATCC 700603, was performed.

Antimicrobial susceptibility testing revealed distinct functional contributions of the individual porins: complementation with functional *ompK35* significantly reduced the cefiderocol MIC for KP5663 from 128 mg/L to 8 mg/L. In contrast, complementation with *ompK36* alone left the MIC unchanged at 128 mg/L (Table 1). This indicates that *ompK35*, rather than *ompK36*, likely serves as the primary non-specific channel for cefiderocol entry in *K. pneumoniae*. The previous inhibitor experiments demonstrated that simultaneous inhibition of NDM-5 and SHV-248 reduced the cefiderocol MIC to 0.25 mg/L. These results confirm that porin deficiency alone is insufficient to confer resistance, but its combined action with NDM-5 and SHV-248 is essential for the development of high-level resistance.

## DISCUSSION

As the first approved siderophore cephalosporin, cefiderocol represents a valuable therapeutic option against MBL-producing Enterobacterales (25). However, in China, where the drug has not yet been officially marketed, cases of intrinsic resistance to cefiderocol in MBL-producing strains have been frequently reported (12, 26, 27). Therefore, in the challenging context of slow novel antibiotic development and escalating resistance, a deeper understanding of the specific mechanisms of cefiderocol resistance mediated by MBLs and other emerging β-lactamases is crucial for curbing the rise in resistance rates, promoting precise anti-infective therapy, and improving patient outcomes (25).

Although cefiderocol exhibits relatively high stability against most β-lactamases, studies have shown that different β-lactamases can still hydrolyze it with varying efficiencies. PER-type β-lactamase-producing *Acinetobacter baumannii* strains with cefiderocol MICs > 4 mg/L have been reported in Russia and Turkey (28). Furthermore, Fröhlich et al. demonstrated that CMY-2 and CTX-M-15 could increase cefiderocol resistance, with further elevations upon acquiring mutations (29). SHV, a core chromosomal gene in *K. pneumoniae*, has been confirmed by several recent studies to be involved in cefiderocol resistance. The novel SHV variant, *bla*_SHV-248_, identified in this study, is closely related to the clinically common *bla*_SHV-11_, suggesting it may have evolved from the latter. Molecular docking simulations indicated its higher affinity for cefiderocol compared to SHV-12 and SHV-11, while nitrocefin hydrolysis assays further confirmed its basal hydrolytic activity to be 1.6-fold higher than SHV-12, indicating that SHV-248 is a variant with enhanced hydrolytic efficiency. We noted that the cefiderocol MIC for *bla*_SHV-12_ expressed in DH5α in this study (0.016 mg/L) was lower than that reported by Lan et al. (0.5 mg/L) (25), a discrepancy potentially attributable to differences in expression vectors and induction conditions. Therefore, we cloned both *bla*_SHV-248_ and *bla*_SHV-12_ into the same expression vector and expressed them in the same host under identical conditions to ensure comparability. Although the expression of *bla*_SHV-248_ alone only slightly increased the cefiderocol MIC, we observed that its basal expression level in the original KP5663 strain was higher than in the recombinant system, which might partly explain its greater contribution to resistance in the clinical strain. This alerts us to the potential threat posed by such novel variants arising from mutations in common SHV types. They may possess higher hydrolytic activity and, if high expression is achieved through promoter mutations or gene amplification, could pose a significant clinical resistance risk, as has been demonstrated for other SHV variants (17, 30). The discovery of *bla*_SHV-248_ in this study further expands the members of the SHV family that can mediate cefiderocol resistance and highlights their potential threat. Notably, although *bla*_SHV-248_ expression alone was insufficient to confer clinical resistance, its higher hydrolytic efficiency compared to SHV-12 makes it a key factor in KP5663.

In recent years, declining cefiderocol susceptibility driven by metallo-β-lactamases has gradually garnered attention. Recent studies have confirmed that NDM-1 and NDM-5 can effectively hydrolyze cefiderocol, while other MBLs like VIM and IMP exhibit lower hydrolytic efficiency (31). Our findings support this conclusion: the metal ion chelator EDTA significantly reduced the cefiderocol resistance of KP5663. Meanwhile, both cloning expression and plasmid conjugation experiments showed that NDM-5 increased the cefiderocol MIC by 32–62.5-fold. However, the manifestation of this hydrolytic activity as a clinical resistance phenotype depends on synergistic effects and genetic background. We observed that when *bla*_NDM-5_ was introduced into hosts lacking other significant β-lactamases (e.g., *E. coli* DH5α and J53), the cefiderocol MIC only increased to 1 mg/L (susceptible range). In contrast, introducing *bla*_NDM-5_ into *K. pneumoniae* ATCC 700603, which endogenously expresses *bla*_SHV-18_, significantly increased the MIC from 0.25 mg/L to 8 mg/L, reaching the resistance level. This led us to a conclusion similar to that of Yang et al.: although NDM has some hydrolytic capacity against cefiderocol, its routine expression typically requires synergy with other mechanisms (e.g., other β-lactamases, iron uptake system mutations) to trigger clinical resistance (24). Notably, although the expression level of *bla*_NDM-5_ appeared higher in the ATCC 700603 recombinant than in KP5663—a difference attributable to the heterologous expression system—the resulting MIC (8 mg/L) was far lower than that of KP5663 (128 mg/L). This indicates that the combined action of NDM-5 and SHV-type enzymes constitutes only the basic level of resistance. The high-level resistance observed in KP5663 suggests the involvement of other critical factors that significantly enhance this combined effect.

In *K. pneumoniae* KP5663, aside from the confirmed absence of mutations in key siderophore uptake-related genes, we noted a truncation of the *ompK35* gene and a complete absence of *ompK36. ompK35* and *ompK36* are the major non-specific porins in *K. pneumoniae*, and their loss has been widely documented as an important mechanism contributing to resistance against β-lactam antibiotics (32). Such deficiencies often coexist with ESBLs, AmpCs, or carbapenemases, synergistically leading to extensively drug-resistant or even pan-drug-resistant phenotypes (32, 33). Cefiderocol possesses a unique dual transmembrane penetration mechanism: its siderophore side chain facilitates active transport via the bacterial iron uptake system, while its C-7 side chain allows passive diffusion through traditional porin channels (34, 35). Although this “Trojan horse” strategy can effectively circumvent certain resistance mechanisms caused by efflux pump overexpression or porin deficiency (36), recent studies suggest that porin deficiency, particularly in the presence of multiple β-lactamases, can still lead to cefiderocol resistance. Moon and Huang reported cefiderocol resistance in a strain harboring multiple SHV enzymes alongside *ompK35* deficiency (16). Another study investigating 14 cefiderocol-resistant CRE isolates also identified the combination of β-lactamase production (including NDM) and porin deficiency as a primary resistance mechanism (37).

Our findings further support this view. In *K. pneumoniae* KP5663, complementation with functional *ompK35* restored susceptibility to cefiderocol, reducing the MIC by 16-fold, whereas complementation with *ompK36* alone showed no significant effect on the MIC. This indicates that the loss of ompK35, but not *ompK36*, contributes to the resistance phenotype and, in combination with multiple high-hydrolysis-efficiency β-lactamases, can lead to high-level resistance. We hypothesize that when both siderophore transport and porin function are intact, the drug can rapidly and abundantly enter the periplasmic space, overwhelming the hydrolytic or efflux capacity of enzymes like SHV-248 and NDM-5, thereby preventing effective resistance (38). However, when *ompK35* is deficient, the passive diffusion pathway is obstructed, slowing the rate of drug concentration increase in the periplasm. This provides ample time for high-hydrolysis-efficiency β-lactamases like SHV-248 and NDM-5 to fully exert their hydrolytic activity, maximizing their contribution and ultimately resulting in high-level resistance.

Our study has several limitations. Due to constraints in our experimental platform, we were unable to purify SHV-248 and determine its enzymatic kinetic parameters against cefiderocol. The assessment of its hydrolytic potential therefore relied on indirect evidence, including its higher activity against the surrogate substrate nitrocefin compared to SHV-12, molecular docking predictions, and distinct phenotypic contributions in heterologous expression assays. While this convergent evidence indicates that SHV-248 possesses heightened hydrolytic potential, future studies with purified enzyme kinetics are required to definitively establish its catalytic efficiency against cefiderocol. Furthermore, we cannot exclude the possibility that complementation of the *ompK35* and *ompK36* genes using an inducible expression system may have resulted in expression levels exceeding those in wild-type strains. However, this does not invalidate the key qualitative finding that restoration of functional *ompK35*—but not *ompK36*—significantly reversed resistance, underscoring the specific and critical role of *ompK35* in the cefiderocol resistance of KP5663.

In summary, this study elucidates a novel and clinically significant resistance pathway in NDM-producing *K. pneumoniae*, which is driven by a streamlined combination of key determinants rather than by the accumulation of multiple β-lactamases. While prior research by Moon and Huang has shown that cefiderocol resistance can arise from a high burden of β-lactamases coupled with porin deficiency (16), we demonstrate that a more minimalist genetic constellation is sufficient: the co-presence of a transmissible *bla*_NDM-5_ gene, a chromosomally encoded novel SHV variant (SHV-248) with heightened hydrolytic potential—both expressed at typical levels—and the common loss of *ompK35*. Critically, the significance of SHV-248 lies not in its standalone potency but in its contextual role. Within a genetic background already containing the potent NDM-5 and a critical permeability defect (ΔOmpK35), the additional hydrolytic burden imposed by SHV-248, though modest, becomes the decisive factor that elevates resistance from moderate to clinically recalcitrant levels. This delineates a distinct “key determinant combination” model for cefiderocol resistance, which operates independently of siderophore-system mutations. Its epidemiological relevance is underscored by the convergence of three prevalent factors: highly transmissible *bla*_NDM-5_ plasmids, the continuous evolution of core chromosomal *bla*_SHV-248_, and frequent porin inactivation. Therefore, beyond reporting a new SHV variant, our findings advocate for an expanded framework in antimicrobial surveillance. For the growing population of NDM-producing *K. pneumoniae*, routine assessment should incorporate porin status—particularly of *ompK35*—alongside β-lactamase profiling to better anticipate and mitigate cefiderocol treatment failures.

## Data Availability

The *Klebsiella pneumoniae* strain KP5663 used in this study has been deposited in the NCBI BioSample database under accession number SAMN52642893.

## SUPPLEMENTAL MATERIAL

Fig. S1 spectrum.00026-26-s0001.tif Nucleotide sequence alignment of wild-type and mutant ompK35.

Fig. S2 spectrum.00026-26-s0002.tif Phylogenetic tree based on SNPs, with SHV-248, SHV-1, SHV-11, and SHV-12 highlighted with red boxes.

Fig. S3 spectrum.00026-26-s0003.tif Genetic context of pGDQ8D112M-NDM, pKP5663-NDM, and pAR8416.

Supplemental material spectrum.00026-26-s0004.docx Supplemental figure legends.

ASM does not own the copyrights to Supplemental Material that may be linked to, or accessed through, an article. The authors have granted ASM a non-exclusive, world-wide license to publish the Supplemental Material files. Please contact the corresponding author directly for reuse.
