# Supplementary figures and images for: Combinatorial effects of a novel SHV-248 variant, NDM-5, and ompK35 deficiency drive high-level cefiderocol resistance in Klebsiella pneumoniae
Source: Microbiol Spectr. 2026 Apr 30;14(6):e00026-26. doi: 10.1128/spectrum.00026-26 (PMC13228031; doi:10.1128/spectrum.00026-26)

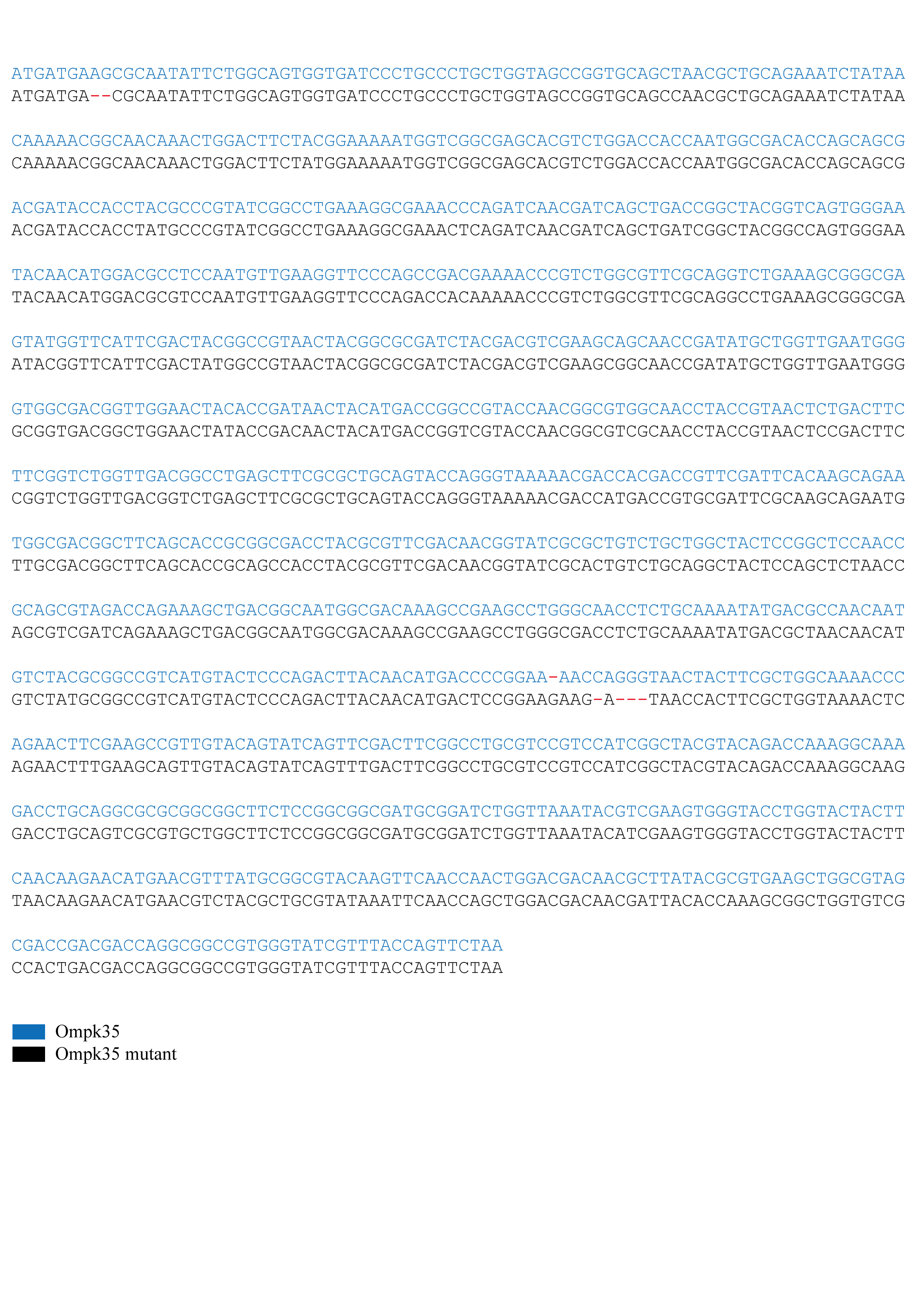

Supplement: Fig. S1 — Nucleotide sequence alignment of wild-type and mutant ompK35. [file spectrum.00026-26-s0001.tif]

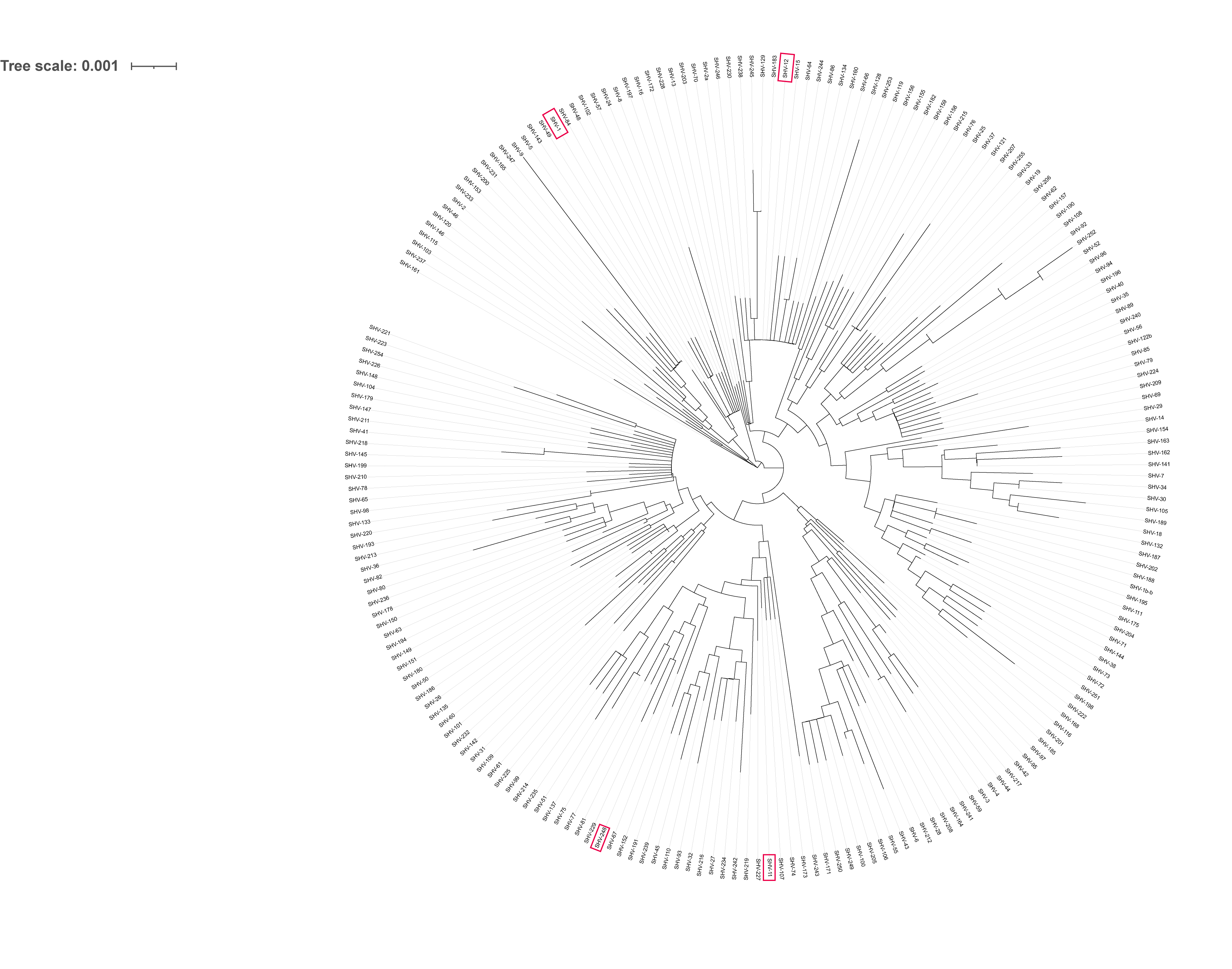

Supplement: Fig. S2 — Phylogenetic tree based on SNPs, with SHV-248, SHV-1, SHV-11, and SHV-12 highlighted with red boxes. [file spectrum.00026-26-s0002.tif]
