# Supplementary material for: Combinatorial effects of a novel SHV-248 variant, NDM-5, and ompK35 deficiency drive high-level cefiderocol resistance in Klebsiella pneumoniae
Source: Microbiol Spectr. 2026 Apr 30;14(6):e00026-26. doi: 10.1128/spectrum.00026-26 (PMC13228031; doi:10.1128/spectrum.00026-26)
Supplement: Supplemental material — Supplemental figure legends. [file spectrum.00026-26-s0004.docx]

**Figure 1S.** Nucleotide sequence alignment of wild-type and mutant *ompK35*. The wild-type sequence is shown in blue, and the mutant sequence in black.

**Figure 2S.** Phylogenetic tree based on single nucleotide polymorphisms (SNPs), with SHV-248, SHV-1, SHV-11, and SHV-12 highlighted with red boxes.

**Figure 3S.** Genetic context of pGDQ8D112M-NDM, pKP5663-NDM, and pAR8416. (A) Circular map comparison of pGDQ8D112M-NDM, pKP5663-NDM, and pAR8416; (B) Linear comparison of pGDQ8D112M-NDM, pKP5663-NDM, and pAR8416.
